# Cylindrical Bidirectional Strain Sensors Based on Fiber Bragg Grating

**DOI:** 10.3390/ma15155399

**Published:** 2022-08-05

**Authors:** Xiaofei Liu, Hui Xie, Haotian Meng, Siqing Zhang, Zifeng Meng

**Affiliations:** 1 School of Safety Engineering, China University of Mining and Technology, Xuzhou 221116, China; 2 Key Laboratory of Gas and Fire Control for Coal Mines, China University of Mining and Technology, Ministry of Education, Xuzhou 221116, China; 3 Logistics Engineering College, Shanghai Maritime University, Shanghai 201306, China

**Keywords:** fiber Bragg grating sensor, bidirectional strain, stress model of the supported surrounding rock, stress–strain relationship

## Abstract

To realize continuous real-time monitoring of the large-scale internal strain field of coal and rock mass, a bidirectional strain sensor based on FBGs encapsulated using a hollow cylindrical steel tube was designed. The sensor’s structural parameters were optimized through unidirectional loading, and the strain change laws of the sensor were analyzed under unidirectional and bidirectional loading conditions, in which the stress-strain fitting curves of the sensor and the relationships of the strain in the vertical and horizontal directions were obtained under different lateral pressure loading conditions. A similar theoretical model was established to verify the accuracy of the linear relationship between the surrounding rock stress and the strain measured by the sensor system.

## 1. Introduction

In recent decades, China has vigorously developed resources. Since the shallow mineral resources have gradually dried up, turning to deep mining has become an inevitable trend. The deep rock mass is a complex environment of “three highs and one disturbance”. The initial geostress was balanced when it was not disturbed by humans or earthquakes. Once artificial projects such as tunnels and mines were carried out, the original stress equilibrium state was broken, subjecting coal and rock masses to solid disturbances that would change the internal stress dramatically, thus, inducing a series of dynamic disasters, such as large deformation of surrounding rock, coal, and rock outburst, and roadway support difficulties [1,2,3]. In addition, during the process of coal mining, stress redistribution occurs in the surrounding rock. Vertical and horizontal stresses change significantly over time, with possible damage in local areas and even induced the collapse of entire roadways. Therefore, research on real-time continuous monitoring technology of the internal damage state of coal and rock mass is important to predict and prevent dynamic disasters such as rock bursts and to improve the accuracy of disaster warning.

When monitoring the shallow surface depth of underground engineering, the empirical analogy method has often been effective. As mining depths increase, ground stress has become more complex and the empirical analogy method has been unable to ensure the safety of actual engineering design; thus, large-scale field measurements must be performed. According to the basic principle of ground stress measurements, first, hydraulic fracturing (HF) in three boreholes in different directions was proposed for measuring three-dimensional stress [4]. Then, it was proposed to calculate three-dimensional ground stress in a borehole based on hydraulic fracturing of primary fractures (HTPF) by using tensile tests of primary fractures in the borehole [5,6], which expanded the feasibility of three-dimensional stress monitoring in a single borehole. In addition, a rock mass’s recovery deformation or strain was measured by artificially releasing its stress, the rock mass’s initial stress was calculated using the known stress–strain relationship, and the two-dimensional stress of the monitoring points was measured [7,8]. Scholars have also used acoustic emission to determine rock mass stress, locate the fracture area, and monitor time and energy [9,10]. However, the requirements for loaded materials are exceptionally high and vulnerable to mechanical noise interference [11,12]. These monitoring methods are all single-point layouts and can only measure the stress of one point. If a wide range of real-time stress or strain needs to be monitored, multiple sets of equipment are needed to drill holes at multiple places simultaneously, and the measurement results are significantly affected by excavation disturbances.

In recent years, optical fiber has developed rapidly. Because of the compatibility between fiber Bragg grating and optical fiber, it was easy to connect multiple fiber Bragg gratings in series through the fiber to realize a wide range of quasi-distributed sensing and to continuously monitor fiber Bragg grating strain or temperature changes in real-time. Therefore, fiber Bragg grating technology has attracted more and more attention from scholars in the world. Meissner et al. from Dresden University [13] buried Bragg gratings in the concrete prism of a bridge to measure the primary linear response under a load and to perform a comparative test with a conventional strain measurement instrument, confirming the feasibility of the application of a fiber Bragg grating sensor. The National Aeronautics and Space Administration [14] installed a fiber Bragg grating sensor network to measure strain and temperature on the space shuttle X-33 for real-time health monitoring. Other scholars have carried out uniaxial compression experiments on rock samples to compare the axial strain of grating and strain gauge, and found that they have the same change rule and that the grating value was significantly better than the strain gauge [15]; a fiber Bragg grating strain sensor was used to measure the circumferential strain of a pipeline to monitor pipeline leakage [16]. Civil facilities [17], bridge structures [18], and pipeline landslides [19] can also be monitored to achieve early detection and positioning of settlement, landslides, erosion, and other events [20]. However, a fiber Bragg grating is relatively fragile; a bare fiber Bragg grating, as a strain sensor, which is directly adhered to the surface of a structure to be tested or buried inside the structure, is very easy to damage in harsh working conditions. Therefore, it needs to be packaged before using [21,22,23,24]; it has mostly been pasted on the surface of the measured object [25] through metal tube packaging [26], capillary steel tube packaging [27], and substrate packaging [28], etc. A fiber Bragg grating sensor has been shown to be suitable for underground coal mine sensing because of its anti-electromagnetic interference, high-temperature resistance, long-term stability, and high radiation resistance [29,30]. The existing fiber Bragg grating sensors have been used to monitor surface strain; surface strain is only the external performance of the internal fracture development of a specimen. The opacity and heterogeneity of a specimen limit the monitoring of the actual strain field. Pre-pouring has also been applied to measured objects to measure internal strain [31]; during the pouring process, a fiber Bragg grating has a low survival rate and does not apply to the geological conditions of coal mines.

Therefore, aiming at the circular borehole in a coal mine, in this paper, we designed a fiber Bragg grating bidirectional strain sensor based on FBG encapsulated by a cylindrical steel pipe to detect the internal strain of coal and rock mass. The theoretical mechanical model of surrounding rock under circular support was established, and the relationships among the stress, strain of surrounding rock, and sensor strain were analyzed. The strain variation law of the sensor under bidirectional loading was analyzed, and the stress-strain fitting curve of the sensor under different lateral pressure loading conditions and the relationship of the strains in vertical and horizontal directions was obtained. The sensor simultaneously monitored the characteristics of transverse stress and axial stress, which provided good technical support for the realization of bidirectional stress monitoring in complex geological environments. In the future, if many sensors can be arranged underground to realize real-time monitoring of stress fields, the accuracy of early warning could be improved.

## 2. Design of the Sensor

**Figure 1:**
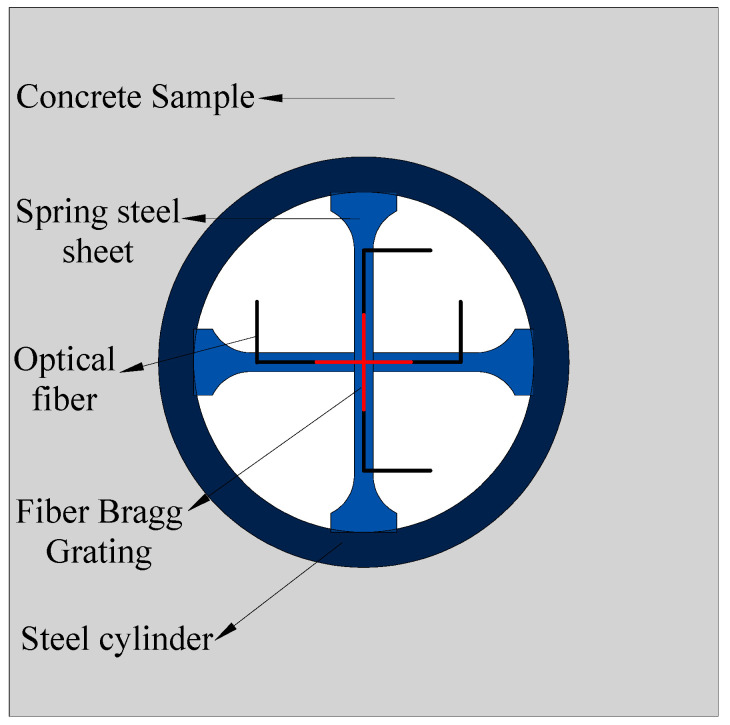
Structure of a cylindrical bidirectional strain sensor based on FBG.

Since the sensor needs to have a certain strength to be used in high-strength coal and rock environments, a steel cylinder was selected as the packaging structure of the fiber Bragg grating sensor. The fiber Bragg grating sensor was fixed on the surface of the spring steel sheet with epoxy resin, and the spring steel sheet was arranged in orthogonal intervals on the longitudinal surface of a cylinder in an orthogonal form (as shown in Figure 1). When the surrounding rock was loaded, using the pouring method with epoxy resin, the deformation of the cylinder bonded closely with the surrounding rock, causing tension or compression of the spring steel sheet. Then, the fiber Bragg grating sensor on the spring steel sheet could monitor the strain of the spring steel sheet, and then the relationship between the stress of the surrounding rock and the strain measured by the sensor is obtained. The “I” type spring steel sheet (wide at both ends and narrow in the middle) was more suitable for the sensor, and therefore, it was selected for this experiment.

Different thicknesses of the cylinder of the fiber Bragg grating sensor and the spring steel sheet affect the range and sensitivity of the sensor. Therefore, it is necessary to determine the appropriate thickness of the cylinder and the spring steel sheet through relevant experiments.

## 3. Stress Model of Surrounding Rock of the Supporting Circular Chamber

The mechanical relationship between the fiber Bragg grating strain and surrounding rock stress was deduced based on elastic and material mechanics. Because the steel cylinder had strong compressive strength, it was considered to have a certain supporting effect on the specimen. Therefore, when the stress analysis of the surrounding rock was carried out, the cylinder was regarded as circular support. When the surrounding rock was supported, the stress was considered to be the superposition of the redistribution stress and the supporting force after excavation.

### 3.1. Elastic Mechanical Characteristics of Roadway Surrounding Rock without Circular Support

**Figure 2:**
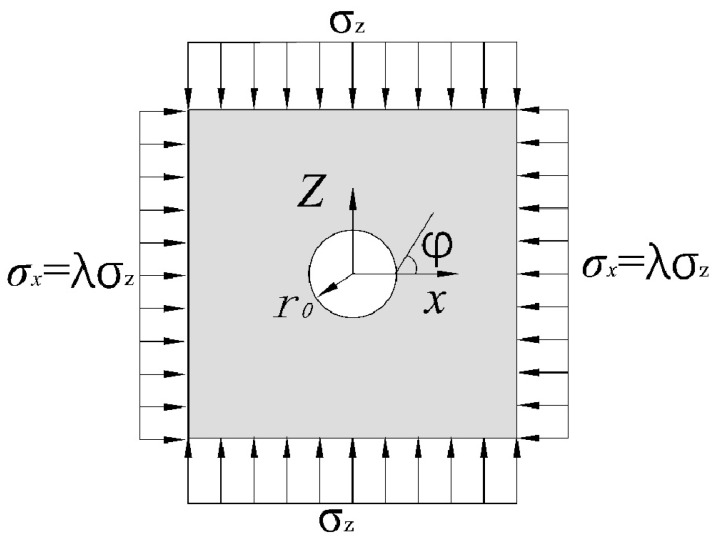
Stress state of roadway surrounding rock without circular support.

A circular chamber with a radius of *r*_0_ was excavated in the surrounding rock. Suppose the horizontal load was symmetrical to the vertical axis and the vertical load was symmetrical to the horizontal axis, the vertical stress was $\sigma_{z}$ and the transverse stress was $\lambda \sigma_{z}$. The stress is shown in Figure 2.

Assuming that the surrounding rock is in an elastic stage, the stress can be calculated by elastic mechanics, thus, the stress at any point in the surrounding rock is:

(1) $$\left\{\begin{array}{c} \sigma_{r1}=\frac{\sigma_{z}}{2}\left[\left(1-\alpha^{2}\right)\left(1+\lambda \right)+\left(1-4\alpha^{3}+3\alpha^{4}\right)\left(1-\lambda \right)cos2\varphi \right]\\ \sigma_{t1}=\frac{\sigma_{z}}{2}\left[\left(1+\alpha^{2}\right)\left(1+\lambda \right)-\left(1+3\alpha^{4}\right)\left(1-\lambda \right)cos2\varphi \right]\;\\ \tau_{rt1}=-\frac{\sigma_{z}}{2}\left(1-\lambda \right)\left(1+2\alpha^{2}-3\alpha^{4}\right)sin2\varphi \end{array}\right.$$

The distance is:

(2) $$\left\{\begin{array}{c} u_{1}=\frac{\sigma_{z}\left(1+\mu \right)}{2E}r_{0}\alpha \left\{\left(1+\lambda \right)+\left(1-\lambda \right)\left[4\left(1-\mu \right)-\alpha^{2}\right]cos2\varphi \right\}\\ \upsilon_{1}=-\frac{\sigma_{z}\left(1+\mu \right)}{2E}r_{0}\alpha \left(1-\lambda \right)\left[2\left(1-2\mu \right)+\alpha^{2}\right]sin2\varphi \end{array}\right.$$

where $\sigma_{r1}$ is radial stress of surrounding rock, kPa; $\sigma_{z}$ is initial ground stress, MPa;$\;r_{0}$ is the original radius of the roadway, m; r is the distance between the surrounding rock and the center of the roadway, m; α is the ratio of $r_{0}$ to r; *λ* is rock side pressure coefficient; φ is the angle in the counterclockwise direction; $\sigma_{t1}$ is the tangential stress of surrounding rock, kPa; $\tau_{rt1}$ is the shear stress of the surrounding rock, kPa; $u_{1}$ is the radial displacement of surrounding rocks, m; *μ* is the Poisson’s ratio of surrounding rocks; *E* is the elastic modulus of surrounding rocks, kPa; $\upsilon_{1}$ is the tangential displacement of surrounding rocks, m.

### 3.2. Elastic Mechanical Characteristics of Roadway Surrounding Rock with Circular Support

**Figure 3:**
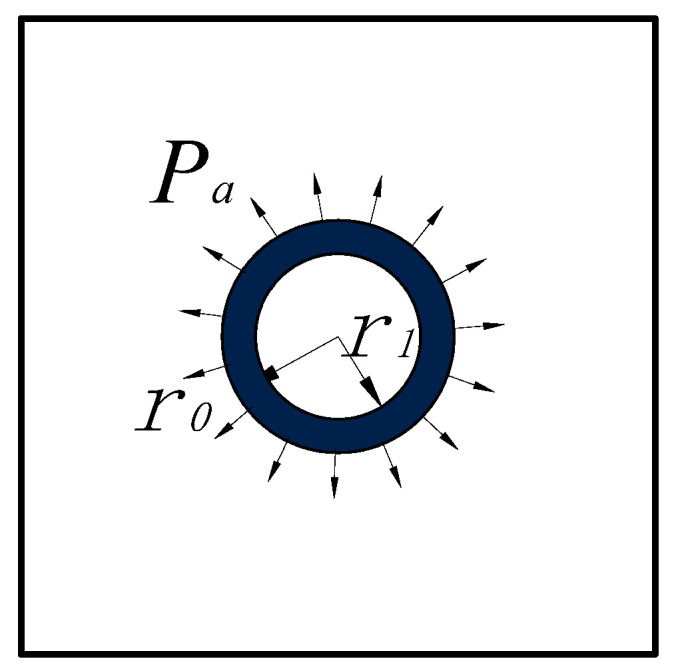
Stress state of the surrounding rock supporting structure.

It was assumed that when the circular chamber was supported immediately after excavation, the surrounding rock had not released stress to produce deformation. Then, the support force model analysis of the support structure on the surrounding rock was shown in Figure 3.

Assuming that there was only supporting force, $p_{a}$, in the surrounding rock, the stress distribution of the surrounding rock was:

(3) $$\left\{\begin{array}{c} \sigma_{r2}=p_{a}\alpha^{2}\\ \sigma_{t2}=-p_{a}\alpha^{2}\\ \tau_{rt1}=0 \end{array}\right.$$

The distance was:

(4) $$\left\{\begin{array}{c} u_{2}=-\frac{\left(1+\mu \right)}{E}p_{a}\frac{r_{0}^{2}}{r}\\ \upsilon_{2}=0 \end{array}\right.$$

At the same time, the supporting structure also bore the role of $p_{a}$. The elastic pressure formula was used to calculate the pressure and displacement of the supporting structure:

(5) $$\left\{\begin{array}{c} \sigma_{r}^{c}=p_{a}\frac{r_{0}^{2}}{r_{0}^{2}-r_{1}^{2}}\left(1-\frac{r_{1}^{2}}{r^{2}}\right)\\ \sigma_{t}^{c}=p_{a}\frac{r_{0}^{2}}{r_{0}^{2}-r_{1}^{2}}\left(1+\frac{r_{1}^{2}}{r^{2}}\right)\\ \upsilon_{r}^{c}=\;\frac{p_{a}\left(1+\mu_{c}\right)}{E_{c}}\frac{r_{0}^{2}}{r_{0}^{2}-r_{1}^{2}}\left[\left(1-2\mu_{c}\right)r+\frac{r_{1}^{2}}{r^{2}}\right] \end{array}\right.$$

where $\mu_{c}\;\mathrm{and}\;E_{c}$ are, respectively, the Poisson’s ratio and elastic modulus of the lining support material, kPa; $r_{0}\;\mathrm{and}\;\;r_{1}$ are, respectively, the outer and inner radius of lining support, m; $\sigma_{r}^{c}\mathrm{and}\;\sigma_{t}^{c}$ are, respectively, the radial stress and tangential stress of supporting structure, kPa; $\upsilon_{r}^{c}$ is the displacement of supporting structure, m; $p_{a}$ is the support, kPa.

### 3.3. Analysis of Mechanical Characteristics of Surrounding Rock of Roadway with Circular Support

**Figure 4:**
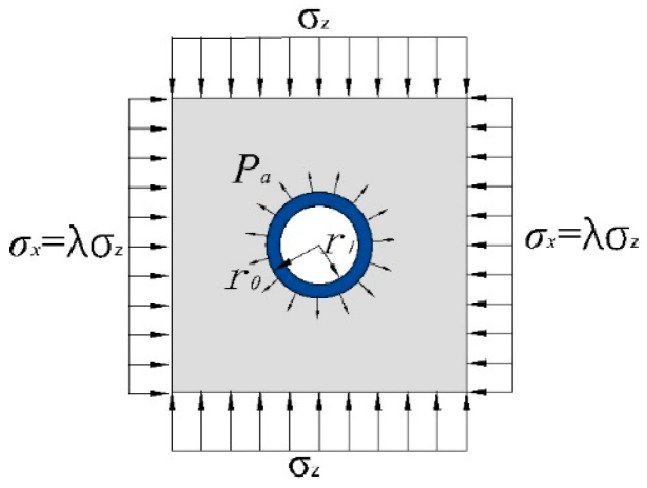
Stress state of surrounding rock of roadway with support.

Surrounding rock stress consists of two parts, one part is the redistribution of surrounding rock stress caused by the excavation of the chamber, and the other part is the supporting resistance, as shown in Figure 4.

In the mining process of coal mines, the axial stress and lateral stress of the surrounding rock in the mining area did not show a linear relationship, the axial stress changed with time, and the lateral stress changes were relatively insignificant. Therefore, in the experimental scheme of this paper, $\sigma_{x}$ (lateral stress) was designed to be a fixed value, $\sigma_{z}$ (axial stress) was a graded load, and $\sigma_{x}=\lambda \sigma_{z}$ was substituted into (1), and (1) and (3) were added to obtain the surrounding rocks stress distribution:

(6) $$\left\{\begin{array}{c} \sigma_{r}=\frac{1}{2}\left(1-\alpha^{2}\right)\left(\sigma_{z}+\sigma_{x}\right)+\frac{1}{2}\left(1-4\alpha^{3}+3\alpha^{4}\right)\left(\sigma_{z}-\sigma_{x}\right)cos2\varphi +p_{a}\alpha^{2}\\ \sigma_{t}=\frac{1}{2}\left(1+\alpha^{2}\right)\left(\sigma_{z}+\sigma_{x}\right)-\frac{1}{2}\left(1+3\alpha^{4}\right)\left(\sigma_{z}-\sigma_{x}\right)cos2\varphi -p_{a}\alpha^{2}\\ \tau_{rt}=-\frac{1}{2}\left(\sigma_{z}-\sigma_{x}\right)\left(1+2\alpha^{2}-3\alpha^{4}\right)sin2\varphi \end{array}\right.$$

Add (2) and (4) to get the displacement of the surrounding rock:

(7) $$\left\{\begin{array}{c} u=\frac{1+\mu }{2E}r_{0}\alpha \left\{\left(\sigma_{z}+\sigma_{x}\right)+\left(\sigma_{z}-\sigma_{x}\right)\left[4\left(1-\mu \right)-\alpha^{2}\right]cos2\varphi \right\}-\frac{\left(1+\mu \right)}{E}p_{a}\frac{r_{0}^{2}}{r}\\ \upsilon =-\frac{\sigma_{z}\left(1+\mu \right)}{2E}r_{0}\alpha \left(1-\lambda \right)\left[2\left(1-2\mu \right)+\alpha^{2}\right]sin2\varphi \end{array}\right.$$

When $\;r\;=r_{0}$, the stress of the surrounding rock should be equal to the pressure $p_{a}$ of the outer radius of the cylinder, that is, the values of (5) and (6) are equal, calculating the supporting force as:

(8) $$\;p_{a}=\frac{r_{0}^{2}-r_{1}^{2}}{2r_{0}^{2}}[(\sigma_{z}+\sigma_{x)}-2\left(\sigma_{z}-\sigma_{x)}cos2\varphi \right]$$

### 3.4. Analysis of the Relationship between the Strain Measured by the Fiber Bragg Grating and the Stress of the Surrounding Rock of the Circular Support

When $r={\;r}_{0}$ (*α* = 1), in the transverse direction (φ=0°) and the displacement of the surrounding rock are:

(9) $$\left\{\begin{array}{c} u_{x}=\sigma_{z}\frac{1+\mu }{2E}r_{0}\left[4-4\mu +\frac{r_{0}^{2}-r_{1}^{2}}{r_{0}^{2}}\right]+\sigma_{x}\frac{1+\mu }{2E}r_{0}\left[4\mu -2-3\frac{r_{0}^{2}-r_{1}^{2}}{r_{0}^{2}}\right]\\ v=0 \end{array}\right.$$

In the axial direction (φ =90°), the displacement of the surrounding rock is:

(10) $$\left\{\begin{array}{c} u_{z}=\sigma_{z}\frac{1+\mu }{2E}r_{0}\left[4\mu -2-3\frac{r_{0}^{2}-r_{1}^{2}}{r_{0}^{2}}\right]+\sigma_{x}\frac{1+\mu }{2E}r_{0}\left[4-4\mu +\frac{r_{0}^{2}-r_{1}^{2}}{r_{0}^{2}}\right]\\ v=0 \end{array}\right.$$

**Figure 5:**
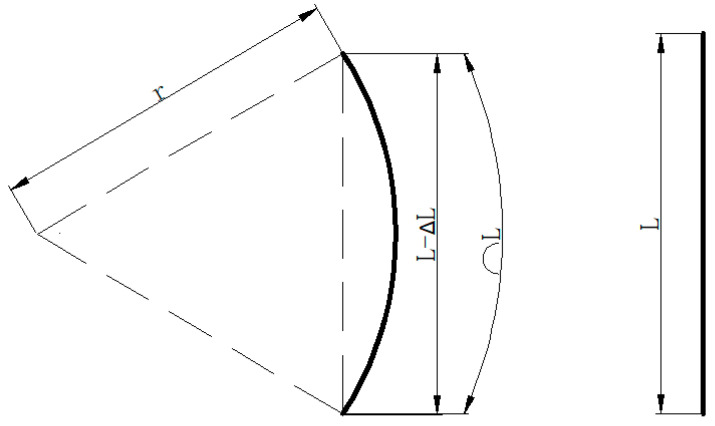
Schematic diagram of the bending deformation of the fiber Bragg grating sensor.

Since there is an intermediate medium between the arranged fiber Bragg grating sensor and the surrounding rock, there is a certain transfer coefficient between the deformation of the fiber Bragg grating sensor and the displacement of the surrounding rock. $\Delta L=ku_{i}$, where k is the transfer coefficient of deformation, ΔL is the deformation of the fibre sensor, and $u_{i}$ is the displacement of the surrounding rock in the direction i. When the fiber Bragg grating sensor bends, a schematic diagram shows the length and radius of the curvature of FBG after bending, as shown in Figure 5.

(11) $$\sin(\frac{L}{2r})=\frac{L-\Delta L}{2r}$$

where L is the original length of the fiber Bragg grating sensor and r is the curvature radius of the fiber Bragg grating sensor.

At this point, the strain of the fiber Bragg grating sensor is:

(12) $$\varepsilon =\pm \frac{h}{r}$$

(13) $$h=r-\sqrt{r^{2}-{\left(\frac{L-\Delta L}{2}\right)}^{2}}$$

where h is the distance between the original center position of the fiber Bragg grating sensor and the center position after bending; “±” represents two opposite bending directions, i.e., 0° and 180°, which correspond to stretching and compressing the central axis of the fiber Bragg grating sensor, respectively [32].

**Figure 6:**
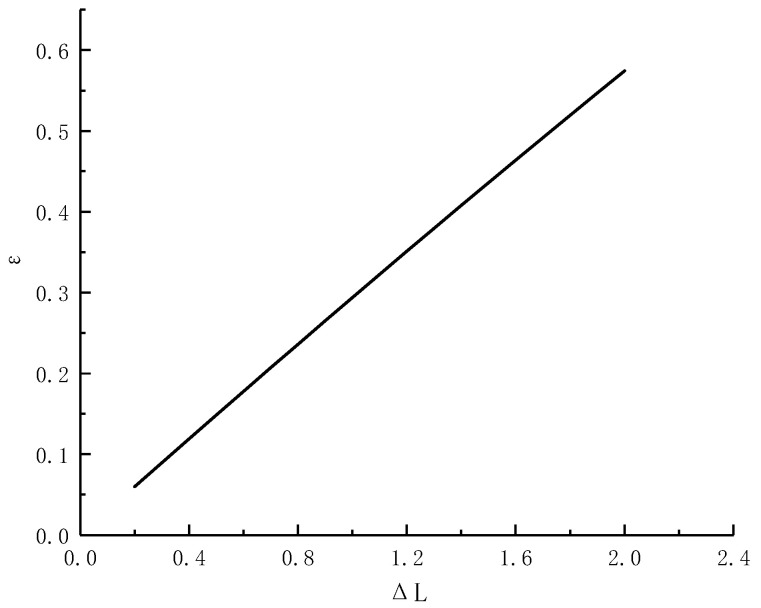
Fitting curve between displacement, Δ*L*, and measured strain, *ε*, produced by grating bending.

According to the most commonly used FBG type, *L* = 10 mm, it was found that the displacement, Δ*L*, generated by grating bending is almost linear with the measured strain, ε, by fitting, as shown in Figure 6. The displacement Δ*L* generated by grating bending was also linear with the displacement $u_{i}$ of surrounding rock. Therefore, it can be concluded that the strain, *ε*, was linear with the displacement, $u_{i}$, of the surrounding rock.

Since *μ*, *β*, *E*, $r_{0}$, and $r_{1}$ are constants, (3)–(9) and (3)–(10) can be expressed as:

(14) $$\left\{\begin{array}{l} \varepsilon_{iz}=\sigma_{z}\lambda_{1}+\sigma_{x}\lambda_{2}\\ \varepsilon_{ix}=\sigma_{z}\lambda_{3}+\sigma_{x}\lambda_{4} \end{array}\right.$$

According to (11), in the elastic stage, the strain measured by the fiber Bragg grating was linearly related to the stress of surrounding rock.

Formula (11) can be converted into:

(15) $$\left\{\begin{array}{l} \sigma_{z}=\frac{\lambda_{4}\varepsilon_{iz}-\lambda_{2}\varepsilon_{iz}}{\lambda_{1}\lambda_{4}-\lambda_{2}\lambda_{3}}=k_{1}\varepsilon_{iz}+k_{2}\varepsilon_{ix}\\ \sigma_{x}=\frac{\lambda_{3}\varepsilon_{iz}-\lambda_{1}\varepsilon_{iz}}{\lambda_{2}\lambda_{3}-\lambda_{1}\lambda_{4}}=k_{3}\varepsilon_{iz}+k_{4}\varepsilon_{ix} \end{array}\right.$$

According to (15), when the constant $k_{i}$ and fiber Bragg grating strain $\varepsilon_{i}$ were known, the axial stress and transverse stress of surrounding rock can be obtained; $k_{i}$ is a constant related to constant $\lambda_{i}$, and $\lambda_{i}$ and $\varepsilon_{i}$ can be obtained through experimental data.

## 4. Experimental System and Scheme

### 4.1. Sample Parameters

The specific parameters of concrete samples and sensors were shown in Table 1.

### 4.2. Experimental System

**Figure 7:**
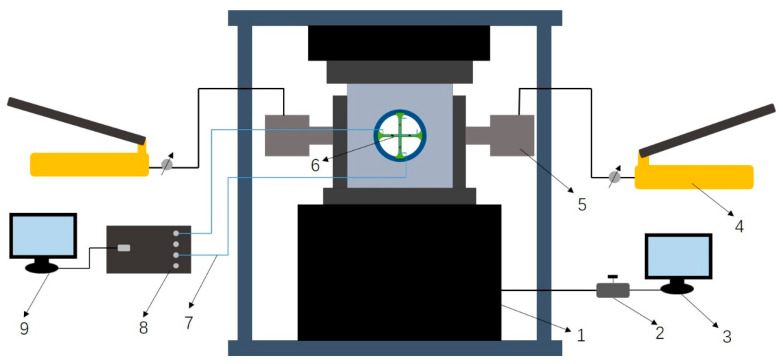
Experimental system diagram. The test system was a combination of many devices, as follows: 1, servo-controlled hydraulic machine; 2, valve; 3, servo-hydraulic machine control system; 4, hydraulic manual pump; 5, jack; 6, fiber Bragg grating sensor; 7, optical fiber; 8, SM130; 9, ENLIGHT System.

The axial loading system used in the test, which adapted a SANS microcomputer control electro-hydraulic servo pressure tester, consisted of a hydraulic oil pump, DCS controller, and Power Test V3.3 control program, as shown in Figure 7. The maximum load of the oil pump could reach 3000 kN, achieving uniform load control and displacement control. The experimental force display resolution (FS) was 1/300,000, and the relative error of the test force indication was 1%. The horizontal load was pressurized with a CP-700 hydraulic manual pump and an FPY-501 thin jack.

The fiber Bragg grating (FBG) strain acquisition system consisted of FBG sensors, an FBG demodulator, and a computer. The sensors packaged were single-point double FC connector sensors, convenient for connecting with another joint after the side of one joint was broken. The fiber Bragg grating center wavelength was 1550.5 nm, the reflectivity was ≥90%, and the length was 10 nm. An SM130 Optical Sensing Interrogator from the Micron Optics company (MOI) was adopted; the scanning frequency was 1000 Hz, the wavelength range was 1510–1590 nm, and the dynamic range was 25 dB. The software ENLIGHT developed by MTO, which was used in conjunction with the demodulator and optimized the optical characteristics of the sensors, monitored the installed sensor system; collected, displayed, and analyzed the data from thousands of FBG sensors at the same time; and converted the optical wavelength parameters into strain, temperature, acceleration, and pressure.

## 5. Sensor Parameter Optimization Experiment

### 5.1. Experimental Scheme

The experimental axial loading scheme was graded loading, with a loading rate of 0.15 kN/s, a grade of 15 kN, and each grade was maintained for 2 min. After loading to 195 kN, it was loaded at 0.15 kN/s until failure. Horizontal transverse stresses were 0 MPa, 2 MPa, 4 MPa, and 6 MPa. Through the uniaxial loading scheme with transverse stress of 0 MPa and the strain value analysis of sensors with different thickness combinations, the optimal sensor model was selected and used in the subsequent bidirectional loading test.

### 5.2. Calibration Experiment

**Figure 8:**
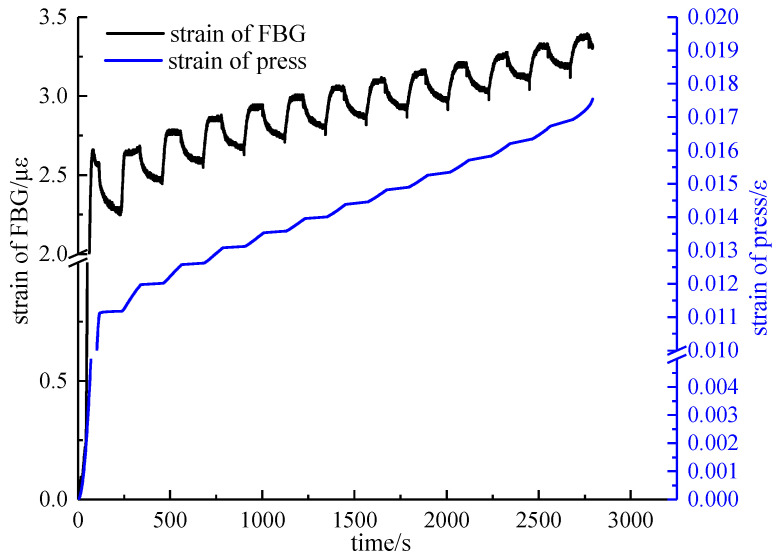
Time-strain curves of the fiber Bragg grating sensor and the press.

Comparing the time-strain curves of the FBG and the press, as shown in Figure 8, it was found that the fitting degree of the two was very high, forming a certain proportional relationship, which was consistent with the theory that there was a strain transfer coefficient. Moreover, it can be seen that the strain value increased rapidly, and then entered the steady growth phase with time, corresponding to the compaction phase and the elastic phase. At each force holding phase, the strain value increased quickly, and then slowly, but still higher than the previous force, and the strain value increased rapidly, and then slowly during the loading phase.

### 5.3. Determination of Optimum Thickness Combination

**Figure 9:**
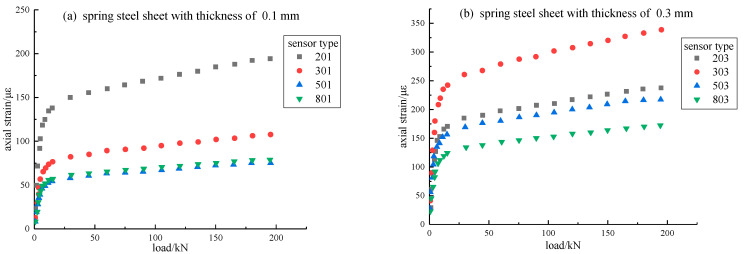

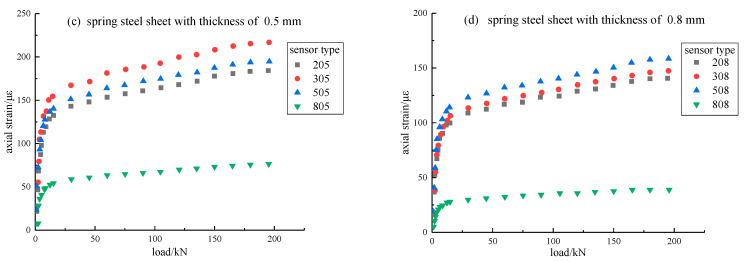
Relationship between combined load and strain with different thicknesses.

The thickness of the external steel cylinder and the thickness of the spring steel sheet can affect the range and sensitivity of the fiber Bragg grating sensor. Therefore, the most suitable thickness of the external cylinder and the thickness of the spring were experimentally determined. Before the experiment, the uniaxial compression test of solid concrete was carried out. The compressive strength was 278.72 kN, and the compressive strength reached or approached 12 MPa. Since the fiber Bragg grating sensor was damaged, the measured strain value was wrong, and it was necessary to ensure that the stress on the fiber Bragg grating sensor did not exceed its elastic range. Therefore, only the experimental data in the loading period of 0–195 kN was used for analysis and comparison. To simplify the analysis process, only the longitudinal strain of the sensor under uniaxial loading were analyzed. The experimental data processing results are shown in Figure 9; each sensor type is assigned a number, in which the first number represents the thickness of the outer cylinder, and the last two numbers represent the thickness of the spring sheet.

According to Table 2, it can be seen that the strain value measured by the 303 sensor (i.e., the thickness of the cylinder was 3 mm and the thickness of the spring sheet was 0.3 mm) was the largest, indicating that the 303 sensor had a relatively high strain transfer coefficient in the designed thickness combination sensor.

Table 3 shows the maximum failure load of concrete samples with different thicknesses of sensors. It could be concluded that when the thickness of the cylinder was relatively thin, the compressive strength of the concrete model with encapsulated sensors was smaller than that of the solid concrete. When the thickness of the cylinder increased to a certain extent, the addition of a steel cylinder would significantly increase the compressive strength of the concrete sample. Thus, the 303 sensor was used for later experiments. Because the larger the strain value was under the same loading force, the more sensitive the sensor was to the loading force, the strain value would be very sensitive when the surrounding rock or the sample was unstable, showing the corresponding abnormal strain.

### 5.4. Bidirectional Loading Experiment

**Figure 10:**
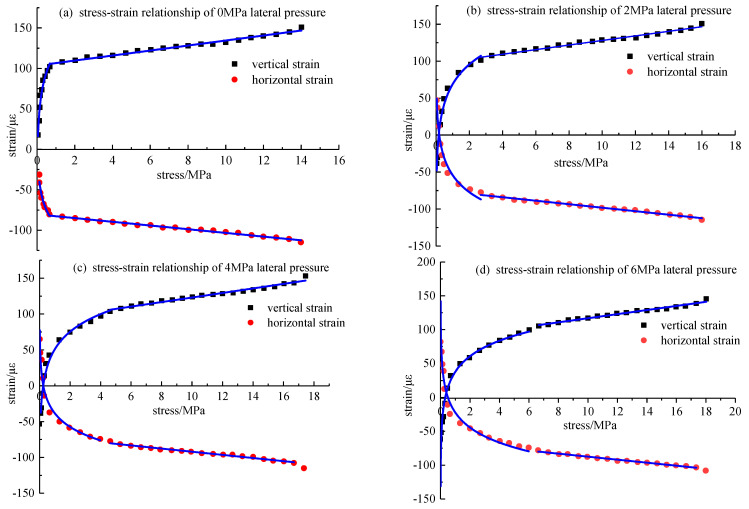
Vertical and horizontal stress–strain relationship under different lateral pressure conditions.

A roadway is not only subjected to the vertical pressure of overburden but also the lateral pressure from surrounding rock in actual work. Therefore, when the two main directions were loaded simultaneously, the vertical and horizontal stress and strain response laws were considered to obtain the stress-strain relationship equations in the vertical and horizontal directions when different lateral pressures were applied. The stress-strain values at the grading loading points of the three sets of experimental data were averaged to fit the stress-strain curves in the vertical and horizontal directions under the condition of two-way loading, as shown in Figure 10.

It can be seen from the figures that applying the lateral pressure caused the sensor to have an initial strain value. When the press started to press, the strain value changed rapidly and entered the steady growth stage. When the lateral pressure was 2 MPa, the stress in the vertical direction of the concrete model was greater than 2 MPa, the strain value entered the steady growth stage, and similar results were obtained when the side pressure was 4 MPa and 6 MPa. Under the same stress, the greater the lateral pressure, the smaller the strain value of the concrete model. In the elastic stage, the intercepts of the vertical and horizontal stress-strain fitting equations decreased gradually as the lateral pressure increased.

By fitting the stress and strain in the vertical and horizontal directions, the relationship equations were obtained, as shown in Table 4.

After applying the lateral pressure, the compressive strength of the packaging sensor concrete model was enhanced as compared with that without the lateral pressure, and the compressive strength increased with an increase in the lateral pressure. Regardless of the applied lateral pressure, the slope of the fitting equation of strain and stress in the vertical direction was about 3.1, and the slope of the proper equation of strain and stress in the horizontal direction was about −2.5, indicating that the slope of the fitting equation was related to the physical properties of the measured surrounding rock, and was not affected by the applied lateral pressure, that is, in a specific surrounding rock environment, the slope of the stress-strain relationship equation measured by the sensor was almost certain.

Due to the existence of microcracks in the specimen, the specimen must go through a compaction stage, and then an elastic deformation stage during the loading process, and there is the strain when entering the elastic stage. Therefore, the initial strain of the elastic stage when only axial loading is used as the initial strain of the elastic stage is as follows:

(16) $$\left\{\begin{array}{c} \varepsilon_{iz}=\sigma_{z}\lambda_{1}+\sigma_{x}\lambda_{2}+\varepsilon_{0z}\\ \varepsilon_{ix}=\sigma_{z}\lambda_{3}+\sigma_{x}\lambda_{4}+\varepsilon_{0x} \end{array}\right.$$

Formula (16) can also be expressed as:

(17) $$\left\{\begin{array}{c} \sigma_{z}=\frac{\lambda_{4}\varepsilon_{iz}-\lambda_{2}\varepsilon_{ix}-(\lambda_{4}\varepsilon_{0z}-\lambda_{2}\varepsilon_{0x})}{\lambda_{1}\lambda_{4}-\lambda_{3}\lambda_{2}}=k_{1}\varepsilon_{iz}+k_{2}\varepsilon_{ix}+k_{0z}\\ \sigma_{x}=\frac{\lambda_{3}\varepsilon_{iz}-\lambda_{1}\varepsilon_{ix}-(\lambda_{3}\varepsilon_{0z}-\lambda_{1}\varepsilon_{0x)}}{\lambda_{3}\lambda_{2}-\lambda_{1}\lambda_{4}}=k_{3}\varepsilon_{iz}+k_{4}\varepsilon_{ix}+k_{0x} \end{array}\right.$$

According to the fitting equation of stress and strain in the elastic stage in Table 3, the average value of each $\lambda_{i}$ can be calculated according to Formula (16), and $\lambda_{1}\;$= 3.145, $\lambda_{2}\;$= −1.42, $\lambda_{3}\;$= −2.255, $\lambda_{4}\;$= 1.26, $\varepsilon_{0z}\;$= 103.5, and $\varepsilon_{0x}\;$= −80.39. By substituting the data into Formula (17), $k_{1}\;$= 1.62, $k_{2\;}$= 1.868, $k_{0z}\;$= −21.389, $k_{3}\;$= 2.97, $k_{4}\;$= 4.138, and $k_{0x}\;$= 25.57, the linear relationship between the surrounding rock stress and the strain measured by the sensor is obtained, as Formula (18):

(18) $$\left\{\begin{array}{c} \sigma_{z}=1.62\varepsilon_{iz}+1.868\varepsilon_{ix}-21.389\\ \sigma_{x}=2.97\varepsilon_{iz}+4.138\varepsilon_{ix}+25.57 \end{array}\right.$$

## 6. Conclusions

For the circular borehole in a coal mine, a fiber Bragg grating bidirectional strain sensor based on cylindrical packaging was designed, which was easy to install and realize multiple sensors in series. The sensor calibration experiment proved that the sensor had good sensing performance. The structural parameters of the sensor were optimized, and the experimental results showed that the strain transfer coefficient of the sensor decreased when the cylinder thickness exceeded a specific value; when the thickness of the cylinder was 3 mm, the average strain measured was the largest. When the thickness of the cylinder was 3 mm and the thickness of the spring steel sheet was 0.3 mm, the strain measured by the sensor was the largest, therefore, the 303 sensor was determined as the final experimental stress sensor. The stress-strain response characteristics of the sensor were analyzed under single and bidirectional loading conditions, and the stress-strain fitting equation of the sensor under different lateral pressure loading conditions was obtained. Based on elastic mechanics, a similar theoretical model was established to verify the accuracy of the linear relationship between the surrounding rock stress and the strain measured by the sensor.

## Figures and Tables

**Table 1:** Experimental parameters.

	Size/mm	Thickness/mm	Material	Elastic Modulus/GPa	Poisson’s Ratio	Density/kg/m^3^
**Concrete model**	150 × 150 × 150	150	Cement, sand, stone	24	0.17	1840
**Cylinder**	150 × Φ75	2, 3, 5, 8	Q235 steel	200	0.3	7850
**Spring steel sheet**		0.1, 0.3, 0.5, 0.8	65 Mn spring steel	190	0.2	7850

**Table 2:** The average strain values of three tests of each sensor type.

	Thickness of Steel Cylinder/mm Thickness of	2	3	5	8	Average Value
Spring Steel Sheet/mm						
0.1		198	109	76	78	115.25
0.3		233	342	222	171	242
0.5		189	184	195	79	161.75
0.8		140	147	157	38	120.5
average value		190	195.5	162.5	91.5	128.75

**Table 3:** Maximum failure load of concrete specimens with different thicknesses of sensors.

Concrete Samples with Different Thicknesses of Sensors	Failure Load/kN
203	214
303	264
503	306
459	459

**Table 4:** Fitting equations of stress and strain in vertical and horizontal directions under different lateral pressures.

Lateral Pressure Value	Direction	Stress-Strain Fitting Equation in Compaction Stage	Stress-Strain Fitting Equation in Elastic Stage
0MPa	vertical direction	$\varepsilon_{1}=34.63ln\sigma +119.12$	$\varepsilon_{9}=3.1\sigma +103.5$
	horizontal direction	$\varepsilon_{2}=-25.35ln\sigma -92.67$	$\varepsilon_{10}=-2.23\sigma -80.39$
2MPa	vertical direction	$\varepsilon_{3}=38.48ln\sigma +70.59$	$\varepsilon_{11}=3.01\sigma +98.03$
	horizontal direction	$\varepsilon_{4}=-33.07ln\sigma -54.13$	$\varepsilon_{12}=-2.36\sigma -74.56$
4MPa	vertical direction	$\varepsilon_{5}=35.1ln\sigma +51.02$	$\varepsilon_{13}=3.17\sigma +91.2$
	horizontal direction	$\varepsilon_{6}=-27.24ln\sigma -38.52$	$\varepsilon_{14}=-2.2\sigma -70.09$
6MPa	vertical direction	$\varepsilon_{7}=33.05ln\sigma +37.7$	$\varepsilon_{15}=3.3\sigma +86.44$
	horizontal direction	$\varepsilon_{8}=-19.89ln\sigma -20.67$	$\varepsilon_{16\;}=-2.23\sigma -65.26$

## Data Availability

The data used to support the findings in this study are available from the corresponding authors upon request.
